# Highly Robust Neutral Plane Oxide TFTs Withstanding 0.25 mm Bending Radius for Stretchable Electronics

**DOI:** 10.1038/srep25734

**Published:** 2016-05-11

**Authors:** Yong-Hwan Kim, Eunji Lee, Jae Gwang Um, Mallory Mativenga, Jin Jang

**Affiliations:** 1 Advanced Display Research Center and Department of Information Display, Kyung Hee University 26 Kyungheedaero, Dongdaemun-gu, Seoul 130-701, Republic of Korea

## Abstract

Advancements in thin-film transistor (TFT) technology have extended to electronics that can withstand extreme bending or even folding. Although the use of ultrathin plastic substrates has achieved considerable advancement towards this end, free-standing ultrathin plastics inevitably suffer from mechanical instability and are very difficult to handle during TFT fabrication. Here, in addition to the use of a 1.5 μm-thick polyimide (PI) substrate, a 1.5 μm-thick PI film is also deposited on top of the TFT devices to ensure that the devices are located at the neutral plane of the two PI films for high folding stability. For mechanical support during TFT fabrication up to the deposition of the top PI film, the PI substrate is spin coated on top of a carrier glass that is coated with a mixture of carbon nanotubes (CNTs) and graphene oxide (GO). The mixture of CNT and GO facilitates mechanical detachment of the neutral plane (NP) TFTs from the carrier glass before they are transferred to a polydimethylsiloxane (PDMS) substrate as islands. Being located in the neutral bending plane, the NP TFT can be transferred to the PDMS without performance degradation and exhibit excellent mechanical stability after stretching the PDMS substrate up to a 25% elastic elongation.

Advancements in thin-film transistor (TFT)-based electronics for display and sensor applications have recently extended to electronics on plastic substrates that can be folded, rolled or crumpled up without cracking the screen or even degrading functionality^1^,^2^,^3^,^4^,^5^,^6^,^7^,^8^,^9^. These deformable electronics can be applied to portable devices or non-flat, curvilinear surfaces in pipelines, automobiles and other related areas. TFT displays with no bezels can be achieved when the driving electronics that are usually located at the peripherals of the display screen (i.e. the bezel) can be folded to occupy the side edges or even back side of the display device. Foldability will also enable easy transportation and storage while not in use. For the final product to be flexible, however, the substrate on which the electronics are built should also be flexible^10^,^11^,^12^,^13^,^14^,^15^. Commonly used flexible substrates are plastics such as polyethylene terephthalate (PET)^16^,^17^, polyethylene naphthalate (PEN), and polyimide (PI)^18^. Thin glass is also used, but rarely, due its inherent fragileness.

Given that typical TFT-based electronic devices have thicknesses less than 1 μm, the thickness of the substrate has dominant contribution on the overall mechanical behavior of the final product. The thinner the substrate, the more bendable and foldable are the overall devices. When the substrate thickness gets to <10 μm, extreme bending becomes possible^19^,^20^,^21^,^22^,^23^. In fact, a 4.8 μm-thick PI substrate has been reported for oxide TFTs, where a bending radius of 2 mm was achieved^24^. However, a bending radius of 2 mm is still very large for foldable electronics. Organic TFTs built on 1 μm-thick PI substrate have also been reported but the smallest bending radius achieved is as large as 2 mm^25^. To decrease the bending radius to less than 1 mm, the TFTs have to be located in a neutral bending plane, similar to what has been reported by Sekitani *et al*., where organic TFTs built on a 12.5 μm-thick PI film can be bent to a radius of 0.1 mm^26^. However, temperature restrictions to less than 100 °C and the use of expensive materials such as gold may not be compatible with inorganic TFTs required for the production of affordable display electronics. Moreover, free-standing ultrathin substrates inevitably suffer from mechanical instability and are very difficult to handle during processing, which necessitates the use of carrier substrates during fabrication.

In this paper, we use ultrathin plastic substrates for extreme bending capabilities, and we also deposit a second ultrathin plastic film of similar thickness on top of the TFT devices to ensure that the devices are located close to the neutral bending plane of the two plastics for minimum strain. Additionally, we also embed a mixture of graphene oxide (GO) and carbon nanotubes (CNTs) into the bottom surface of the ultrathin substrate for mechanical support and reduction of electrostatic discharge (ESD) damage. The material used as the ultrathin substrate and ultrathin top film is a solution processed 1.5-thick PI film, given the many advantages of PI compared to other plastics such as PET or PEN, which include higher glass transition temperature, lower coefficient of thermal expansion (CTE), higher chemical resistance, and higher processing temperature. Consequently, the PI allows fabrication process temperatures as high as 300 °C, which is very important to achieve high performance and highly stable inorganic TFTs. For handling purposes during fabrication, carrier glass, on which the CNT/GO backbone and PI substrate are first deposited from solution by spin coating, is employed (see Fig. 1a–f for schematized fabrication process). TFTs with an amorphous oxide semiconductor, amorphous indium−gallium−zinc oxide (a-IGZO), are employed as the test devices. The a-IGZO is a suitable semiconductor because a-IGZO TFTs exhibit high field-effect mobility (μ_FE_) and low threshold voltage (V_TH_), even when the a-IGZO is deposited at room temperature, making it compatible with flexible substrates^27^,^28^. After fabrication, the TFTs are mechanically detached from the carrier glass (Fig. 1g) and transferred to a polydimethylsiloxane (PDMS) substrate. Mechanical stability of the neutral plane (NP) TFTs is tested by performing bending and stretch tests.

## Results and Discussion

**Figure 1:**
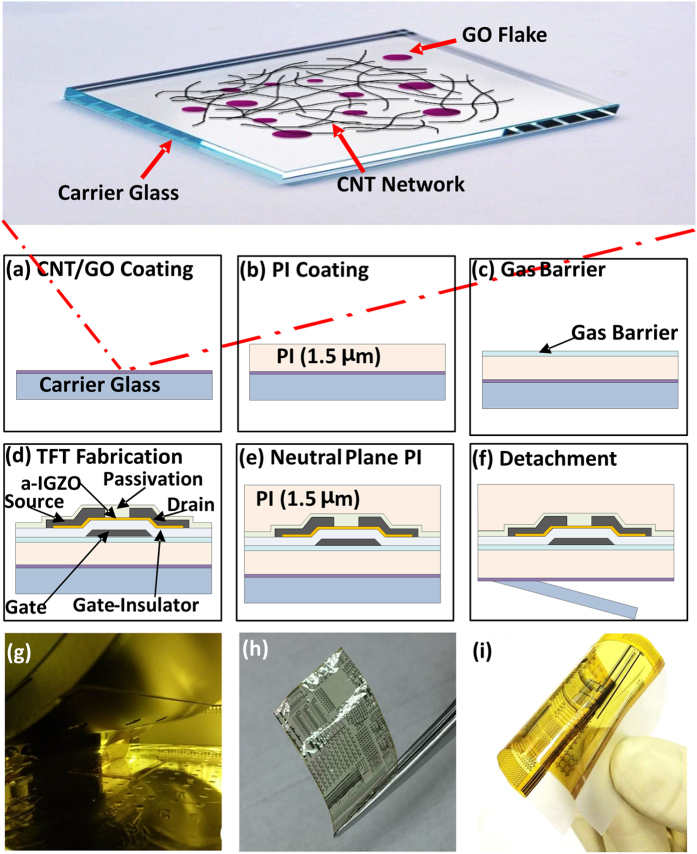
Fabrication process flow of neutral plane TFTs on a 1.5 μm PI substrate. (**a**) Formation of a CNT/GO release layer on carrier glass by spin coating. (**b**) Formation of PI substrate by spin coating. (**c**) Formation of a gas barrier, composed five alternating SiO_2_ and SiN_x_ layers deposited by plasma-enhanced chemical vapor deposition. (**d**) TFT formation. (**e**) Formation of a top PI film (1.5 μm) by spin coating. The presence of the top PI film positions the TFTs close to the neutral bending plane. (**f**) Mechanical detachment from carrier glass. (**g**) Image of the machine used for the mechanical detachment. (**h**) Image of TFT device fabricated on a 1.5 μm PI substrate after detachment from carrier glass. (**i**) Image of TFT device fabricated on a 1.5 μm PI substrate and encapsulated with a 1.5 μm PI layer after detachment from carrier glass.

**Figure 2:**
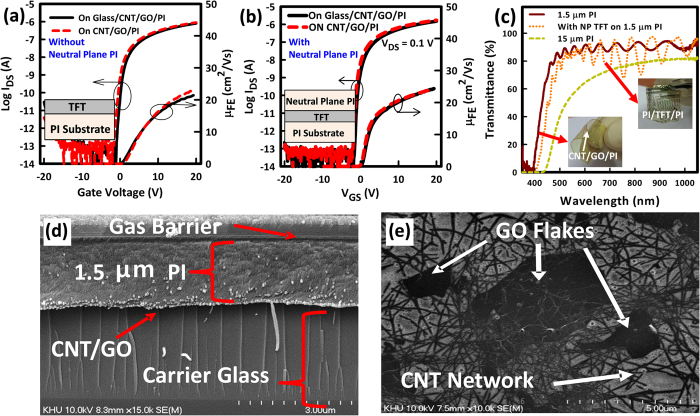
TFT operation and substrate structure. (**a,b**) Transfer characteristics and field-effect mobility, before and after detachment of a-IGZO TFTs (**a**) without and (**b**) with the top neutral plane PI film. (**c**)Transmittance measurements and (insets) photographs of a substrate with and without TFT devices after detachment from carrier glass. (**d**) Cross sectional scanning electron microscope (SEM) image of the substrate before detachment from carrier glass. (**e**) SEM image of the bottom surface of the PI substrate after detachment from carrier glass.

The fabrication process of the a-IGZO NP TFTs is depicted in Fig. 1 and details of the materials can be found in the Methods Section. The oxide TFTs on plastic/glass can be easily detached mechanically, without the use of expensive lasers, because of the existence of the CNT/GO layer^23^. Images of flexible a-IGZO TFT devices, without and with the top NP PI, before transfer to PDMS, are shown in Fig. 1h,i, respectively. The typical performance of a-IGZO TFTs fabricated without and with the top NP PI film are shown in Fig. 2a,b. In each case, TFT characteristics, before and after detachment from the carrier glass, are provided. In both cases, the TFT’s V_ON_ shifts towards the negative gate voltage (V_GS_) direction after detachment. However, the shift is much smaller for the NP TFTs. For TFTs fabricated without the top NP PI film, the μ_FE_ increases from 22 ± 0.2 to 24 ± 0.4 cm^2^/V·s, V_ON_ shifts to the negative V_GS_ direction from −0.8 ± 0.5 to −1.4 ± 0.3 V and SS increases from 340 ± 25 to 371 ± 64 mV/dec after detachment. As for the NP TFTs, the μ_FE_ increases from 22 ± 0.4 to 23 ± 0.5 cm^2^/V·s, V_ON_ shifts to the negative V_GS_ direction from −1 ± 0.2 to −1.4 ± 0.2 V and SS increases from 196 ± 20 to 210 ± 24 mV/dec after detachment.

The negative V_ON_ shift (ΔV_ON_) that occurs after detachment from carrier glass is related to the mechanical strain during the detachment process. Channel conductivity may increase after the application of mechanical strain to the TFT, which may result in a negative shift of V_ON_ after detachment. It is previously reported that mechanical strain causes an increase (in the case of tensile strain) in donor concentration, which causes the Fermi level to shift closer to the conduction band and, therefore, a negative shift of V_ON_^29^. The generated donors remain even at the re-flat state (after bending) because they cannot be rapidly annealed out at room temperature. Optical transparency in the visible range of the 1.5 μm-thick substrate with the CNT/GO backbone is around 90% (Fig. 2c). Transparency of the fabricated TFTs on the 1.5 μm-thick PI substrate is also around 90% and average transmittance decreases to 85% for the NP TFTs (Fig. 2c). Note that transparency decreases to around 80% when the PI thickness increases to around 15 μm (Fig. 2c), as in previous reports^20^. A scanning electron microscope (SEM) image of the substrate before detachment is shown in Fig. 2d. The CNT/GO layer could not be seen from the cross-section by SEM as it is very thin (<1 nm). However, after detachment from glass, the CNT/GO could be seen embedded at the back side of the PI substrate by high resolution SEM (Fig. 2e). Note here that there were no CNT/GO residues left on the carrier glass and that the backside of the PI substrate had a sheet resistance of around 2 kΩ/□. It can be seen from the SEM image that the GO takes a flake-like structure, while the CNT forms links among the flakes. The very thin CNT/GO layer decreases the peel strength of the PI substrate from ~0.4 to 0.1 N/cm^20^. As the CNT/GO layer remains imbedded into the backside of the PI, it acts as its backbone and consequently increases its tensile strength from ~230 to ~240 MPa, lowering the tensile elongation from ~8% to ~4%^20^. The result is a robust standalone plastic substrate that is free from wrinkling or curling. Furthermore, the conductive CNTs minimize electrostatic discharge (ESD) damage, as they may aid in the release of localized ESD^23^. The sample is detached using a special detach-machine (See Fig. 1g), which has a radius of 15 cm to reduce stress on the sample during the detachment process. Because the pull strength is distributed uniformly to the sample, metal cracks are not visible.

**Figure 3:**
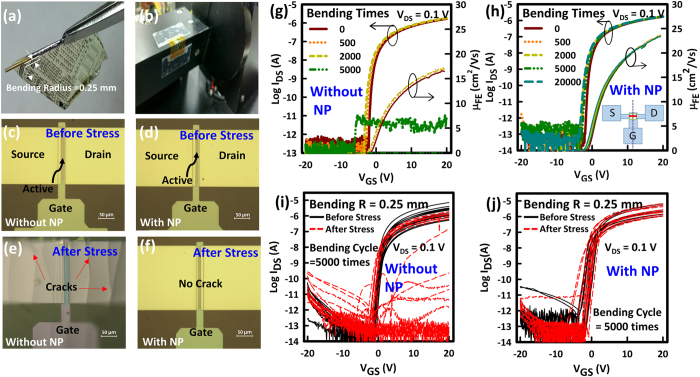
Bending Test: (**a**) Optical micrograph of a sample that is wound on a cylinder with a radius of 0.25 mm. (**b**) Photograph of the extreme bending machine. (**c,d**) Optical images of TFTs (**c**) without and (**d**) with the top PI film before bending. (**e,f**) Optical images of TFTs (**e**) without and (**f**) with the top PI film (neutral plane TFTs) after 5000 bending cycles. (**g,h**) Transfer characteristics as a function of extreme bending cycles of a-IGZO TFTs (**g**) without and (**h**) with the top neutral plane PI film. (**i,j**) Transfer characteristics before and after bending for 5000 times of several a-IGZO TFTs, (**i**) without and (**j**) with the top neutral plane PI film, distributed across a 6 inch by 6 inch substrate. All TFTs have channel width (W) = 50 μm and channel length (L) = 8 μm.

To evaluate bending stability of the a-IGZO TFTs, samples are wound to cylinders of decreasing radius and devices tested while bending. Starting from a cylinder with a radius of 3 mm all the way down to a cylinder with radius of 0.25 mm (Fig. 3a). Devices with and without the top PI film both show negligible changes in operation for all bending radii. Figure 3a shows an image of a sample that is wound to a cylinder with a radius of 0.25 mm. The bending direction is perpendicular to the direction of the TFT current flow and the bending tests are performed with TFTs bent outwards to induce tensile strain. Note here that 0.25 mm is the smallest that could be achieved practically because testing devices, while bending to bending radii <1 mm, is extremely difficult. Given that organic TFTs built on a 12.5 μm PI film by Sekitani *et al*. could be bent to a radius of 0.1 mm, owing to them being located at the neutral bending plane^26^, it is reasonable to believe that devices that are also located at the neutral bending plane but built on a much thinner (1.5 μm) PI substrate should, in principle, be able to withstand bending radii much smaller than 0.1 mm. The organic TFTs by R. Amir *et al*. are built on 1 μm-thick PI but can only withstand minimum bending radius of 2 mm before degradation probably because they are not located in the neutral bending plane. Therefore, in this report, a combination of a thin substrate (1.5 μm) and the location of the oxides TFTs at the neutral bending plane, makes their mechanical stability stands out among all flexible inorganic TFTs in literature.

Young’s modulus (Y) can be used to express the strain (ε) experienced by any TFT device as a function of film radius and thickness as below^11^:


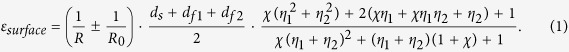


Here, R is the radius of curvature, R_0_ is the radius of curvature of the structure before applying any external bending moment, χ = Y_f_, η_1_ = d_f1_/d_s_, and η_2_ = d_f2_/d_s_. Y_s_ and Y_f_ are, respectively, the Young’s moduli of the substrate and any stiff film that may exist on the top or bottom surface of the substrate. Likewise, d_s_, d_f1_, and d_f2_ are, respectively the thicknesses of the substrate, stiff film on top, and stiff film on the bottom of the substrate. Thus, all other parameters being the same, the strain acting on the TFTs decreases with decreasing substrate thickness^11^. For the cylinder with radius R = 0.25 mm, the corresponding strain applied to the top surface is 0.48%, in the case of TFTs without the top PI film. As for the neutral plane TFTs, the engineering strain, ε_eng_ can be written as


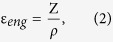


where ρ is the radius of curvature at the neutral plane, varies linearly with distance, z, from the neutral plane (Supplementary Figure S1a)^26^. For the a-IGZO TFTs presented herein, the a-IGZO channel is located 60 nm from the neutral plane, giving ε_eng_ = 0.023%. Having a top PI film thus decreases the strain on the devices by more than 20 times

To further evaluate the effect of the top PI film on the bending stability of the TFTs, the samples are exposed to repeated extreme bending cycles to a radius of 0.25 mm, using a special bending machine (Fig. 3b). The schematic of the extreme bending machine is shown in Supplementary Figure S1b. The placement of the sample on the bending machine is such that the direction of the bending stress is perpendicular to the TFT current flow. The external bending force is applied up to 20,000 times. TFTs without the top NP PI film are bent only up to 2,000 times, before breakdown (Fig. 3g). Interestingly, NP TFTs are still operational even after bending for 20,000 times (Fig. 3h). It can be seen that for the first 2,000 bending cycles, the NP TFTs exhibit better stability compared to the TFTs without the top NP PI. Optical images of TFT devices before and after bending for 5,000 times are respectively shown in Fig. 3c,e for the TFTs without the top PI film. Visible cracks can be seen on the source drain metal contacts after stress. As shown in Fig. 3d,f, there are no visible cracks in the neutral plane TFTs after bending for 20000 cycles. Although the calculated strain for the TFTs that are not in the neutral bending plane turns out small (0.48%) because the substrate is very thin (1.5 μm), it is, however, important to note here that the bending radius is 0.25 mm, which is equivalent to folding. Cracks are, therefore, more likely to occur in the devices without the top PI.

Performance degradation (before breakdown) characterized by negative ΔV_ON_, such as that experienced by the TFTs without the NP PI after detachment from glass (Fig. 2a), has been explained to be the result of tensile strain-induced increase in channel conductivity that is brought about by an increase in the donor concentration^29^. Breakdown, however, is due to crack formation in the electrodes, which can be verified optically (see Fig. 3(e,f)). There is no visible evidence of cracks in TFTs that can still operate, despite them having gone through negative ΔV_ON_. Several TFTs are tested across 6 inch by 6 inch substrates to investigate device uniformity. Figure 3i,j show respectively the results for the TFTs without the NP top PI film and the NP TFTs. After 5000 bending cycles, a large number of TFTs without the top PI film broke down (Supplementary Figure S2a), whereas none of the NP TFTs broke down after 5,000 bending cycles (Supplementary Figure S1b). A negative ΔV_ON_ = ~0.5 V can be estimated from the difference in the means of the distributions of the V_ON_ values before and after stress (Supplementary Figure S2a). For the NP TFTs, the ΔV_ON_ = ~0.25 V and none of them breakdown (Supplementary Figure S2b), proving that the addition of a top PI film that is of the same thickness as the PI substrate does locate the TFTs devices closer to the neutral plane of the two PI films, resulting in improved folding stability.

**Figure 4:**
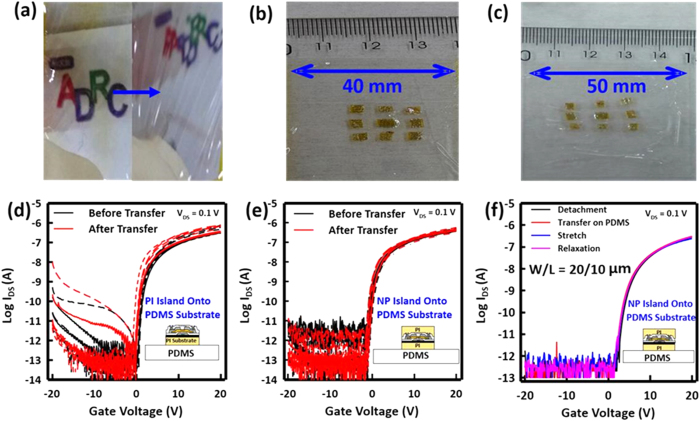
Stretching Test: (**a**) Optical micrograph of PDMS films in the (left) flat and (right) rolled and twisted conditions. (**b,c**) Image of neutral plane TFTs after being transferred to the PDMS film (**b**) before and (**c**) while being stretched in the direction parallel to the TFT channel. Extended length of the PDMS film is 10 mm (25% strain). (**d,e**) Transfer characteristics of a-IGZO TFTs on PI, (**d**) without and (**e**) with the neutral plane PI, before and after transfer onto the PDMS substrate and (insets) schematic of cross sections of the respective TFT structures. (**f**) Transfer characteristics of the neutral plane TFTs measured at various stages of the process.

In addition, the NP TFTs also remain mechanically stable after being transferred to the stretchable PDMS substrate. Here, PDMS is a preferred choice, given that it is transparent and can be rolled, twisted and stretched (Fig. 4a). Note here that the NP TFTs are transferred onto the PDMS film in the form of islands, in order to achieve partial stretchability in the PDMS-only area (Supplementary Figure S3). The NP TFTs are unstretchable. Figure 4b and c show respectively an unstretched and a stretched sample. The direction of the stretch is parallel to the TFT channel. A sample that is initially 40 mm in length is stretched until it is 50 mm long without device degradation, which corresponds to a 25% elastic elongation. TFT performance is also traced during the transfer to PDMS process and compared for TFTs without and with the top PI as shown in Fig. 4d,e, respectively. The results indicate that the NP TFTs are more stable than the TFTs that are not located in the neutral bending plane during the transfer to PDMS (Supplementary Figure S4). The degradation that occurs in the TFTs that are not in the neutral bending plane can be attributed to mechanical stress during the transfer process. On the contrary, the performance of The NP TFTs is almost unchanged (Fig. 4e), and they remain stable after the PDMS is stretched and relaxed (Fig. 4f), indicating that the combinational use of an ultrathin substrate and the location of the TFTs close to the neutral bending plane results high stability under mechanical strain.

## Conclusion

High flexibility and mechanical bending stability has been achieved in oxide TFTs by fabricating them on an ultrathin (1.5 μm) PI substrate and encapsulating them with a PI film of thickness equal to that of the substrate, thereby ensuring their locating close to the neutral bending plane. The PI substrate is first spin coated on top of a carrier glass for mechanical support during TFT fabrication, and then mechanically detached after fabrication. A mixture of CNTs and GO that is inserted between the glass and PI as a release layer, remains embedded to the back side of the PI substrate – further strengthening the devices from curling and ESD damage. There are no visible cracks in the neutral plane (NP) TFTs after bending for 20000 cycles to a radius R = 0.25 mm, indicating that the existence of top PI film does locate the TFTs close to the neutral plane. The high mechanical stability of the NP TFTs allows them to be transferred to a PDMS film, without degradation in performance, to yield a partially stretchable sample. The NP TFTs retain fairly high electrical performance after the substrate is stretched to 25% elastic elongation. Thus, the oxide TFTs in the NP can be used as building blocks for stretchable electronics.

## Methods

### Substrate Preparation

A mixture of a water-based GO solution and CNT solution is spin-coated on standard display glass (6 inch by 6 inch) to a thickness ranging from 0.7 to 1 nm (Fig. 1a). The CNT and GO solutions are acquired from Unidym Carbon Nanotubes and Graphene Supermarket, respectively. The CNT/GO solution is spin-coated for 25 s at 1000 rotations per minute (rpm) followed by subsequent baking at 290 °C in a vacuum oven. The PI is also deposited from solution and is spin-coated at 3500 rpm to achieve a thickness around 1.5 μm (Fig. 1b). An additional baking of the carrier glass/CNT/GO/PI substrate is carried out at 450 °C in N_2_ ambient. The substrate is covered by SiO_2_ and SiN_X_ layers (five layers in total starting with SiO_2_) with thickness of 25 nm each at 300 °C by plasma enhanced chemical vapor deposition (PECVD) to form a buffer layer, which acts as a gas barrier (Fig. 1c).

### TFT Fabrication

The TFT fabrication process is initiated by the deposition of Mo by sputtering on top of the gas barrier and its patterning to form the gate electrode. This is followed by the deposition of a 250 nm thick SiO_2_ layer through PECVD at 350 °C as the bottom gate insulator. Then, a 20 nm thick a-IGZO layer is deposited using a polycrystalline IGZO target (In_2_O_3_: Ga_2_O_3_: ZnO = 1:1:1 mol %) by direct current (dc) sputtering at 200 °C. The sputtering is done in an Ar and O_2_ gas mixture (Ar:O_2_ ratio = 4:16 sccm). The a-IGZO is patterned by an indium tin oxide (ITO) etchant to form the active island. Note that the gate-insulator and the active layer are deposited in a cluster deposition tool, without breaking vacuum to ensure a clean active-layer/gate-insulator interface. A 150 nm thick Mo layer is deposited by sputtering and patterned as the source/drain electrode, followed by the deposition of a 200 nm thick SiO_2_ layer by PECVD at 200 °C as the passivation layer. The schematic cross section of the full TFT devices is shown in Fig. 1d. The samples are annealed at 250 °C in vacuum for 4 h to ensure a reproducible unstressed state. The sample is cut into two and on one half, another PI film (1.5 μm) is deposited from solution on top of the devices (Fig. 1e). This is followed by the formation of contact holes at the probe pads by dry etching. Finally, a detachment process from glass is performed mechanically for the two halves (one with the top PI film and the other without), using a mechanical detach machine, specifically designed for this purpose (Fig. 1f).

### Device Characterization

TFTs are characterized in a black box using an Agilent 4156C precision semiconductor parameter analyzer. TFTs’ current-voltage characteristics are measured in dark and at room temperature. The field-effect mobility (μ_FE_) and subthreshold swing (SS) are extracted from the conventional metal-oxide-semiconductor field effect transistor (MOSFET) equation^30^. The μ_FE_, derived from transconductance (g_M_), ∂I_DS_/∂V_GS_, is given by μ_FE_ = (g_M_L)/(WV_DS_C_OX_), where, I_DS_ is the drain current, V_GS_ is the gate voltage, C_OX_ is the gate insulator capacitance per unit area and V_DS_ = 0.1 V is the drain voltage. The SS is taken as (d log(I_DS_)/dV_GS_)^−1^ of the range 10 pA ≤ I_DS_ ≤ 100 pA, with V_DS_ = 0.1 V. For simplicity, the TFT’s turn-on voltage (V_ON_) is taken as the V_GS_ at which the I_DS_, measured with V_DS_ = 0.1 V, starts to monotonically increase. Surface analyses of the CNT/GO PI substrate was performed using scanning electron microscope (SEM).

The extreme bending machine can bend the device from −120 to 120 degrees. There are clamps which hold the device in place. The clamps are fixed by magnets to avoid damage to the TFTs. The distance between two clamps is 0.5 mm. The number of times and the bending speed can be adjusted through a control box. After bending, the device is released for measurement. The machine can be adjusted to give an exact angle on the sample. As the distance between the two clamps is 0.5 mm, the folded part of the sample forms an arc. Therefore, the bending radius can be extracted from the following equation


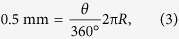


where θ is the bending angle.

### Transfer process onto PDMS film

A de-airing process is first performed on a liquid type PDMS solution (Sylgard 184, 10:1 silicone gel:crosslinker weight ratio, Dow Corning) to remove air bubbles from the solution. After depositing a release layer on a carrier glass, the de-aired PDMS film is spin-coated onto the carrier glass for 30 s at 1200 rpm and cured at 150 °C for 15 min, resulting in 45 μm-thick PDMS film. The standalone strectahble PDMS film is achieved by mechanical detachment of the PDMS from the carrier glass. TFT samples on the PI substrate are cut into islands of varying sizes from 1 mm x 1 mm to 5 mm x 5 mm by using a CO_2_ laser, and transferred onto the PDMS film using PI tape.

## Additional Information

**How to cite this article**: Kim, Y.-H. *et al*. Highly Robust Neutral Plane Oxide TFTs Withstanding 0.25 mm Bending Radius for Stretchable Electronics. *Sci. Rep*. **6**, 25734; doi: 10.1038/srep25734 (2016).

## Supplementary Material

Supplementary Information
