# Supplementary material for: Highly Robust Neutral Plane Oxide TFTs Withstanding 0.25 mm Bending Radius for Stretchable Electronics
Source: Sci Rep. 2016 May 11;6:25734. doi: 10.1038/srep25734 (PMC4863145; doi:10.1038/srep25734)
Supplement: Supplementary Information [file srep25734-s1.pdf]

Supplementary

Highly Robust Neutral Plane Oxide TFTs Withstanding 0.25 mm Bending Radius for Stretchable Electronics

Yong-Hwan Kim[1], *Eunji Lee*[1], *Jae Gwang Um*[1], *Mallory Mativenga*[1], *and Jin Jang**

[1]Advanced Display Research Center and Department of Information Display, Kyung Hee University

26 Kyungheedaero, Dongdaemun-gu, Seoul 130-701, Republic of Korea

Correspondence and requests for materials should be addressed to Jin Jang (email: jjang@khu.ac.kr, phone number: +82-10-3720-6924)

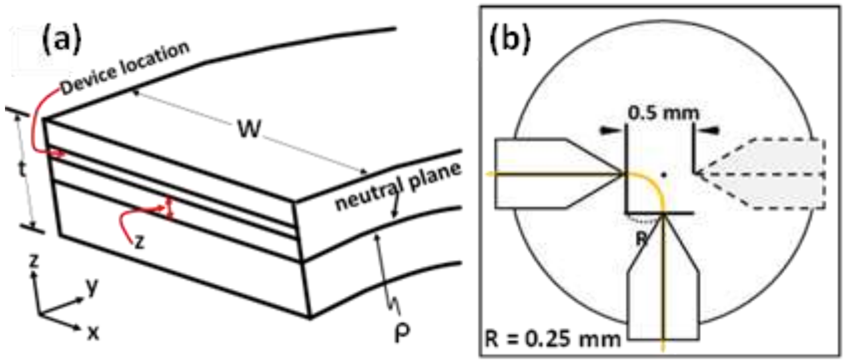

Figure S1. (a) Bending schematic showing the distance z from the neutral plane. (b) Schematic of the extreme bending machine (bending radius = 0.25 mm). The extreme bending machine can bend the device from -120 to 120 degrees. There are clamps which hold the device in place. The clamps are fixed by magnets to avoid damage to the TFTs. The distance between two clamps is 0.5 mm. The number of times and the bending speed can be adjusted through a control box. After bending, the device is released, for measurement. The machine can be adjusted to give an exact angle on the sample.

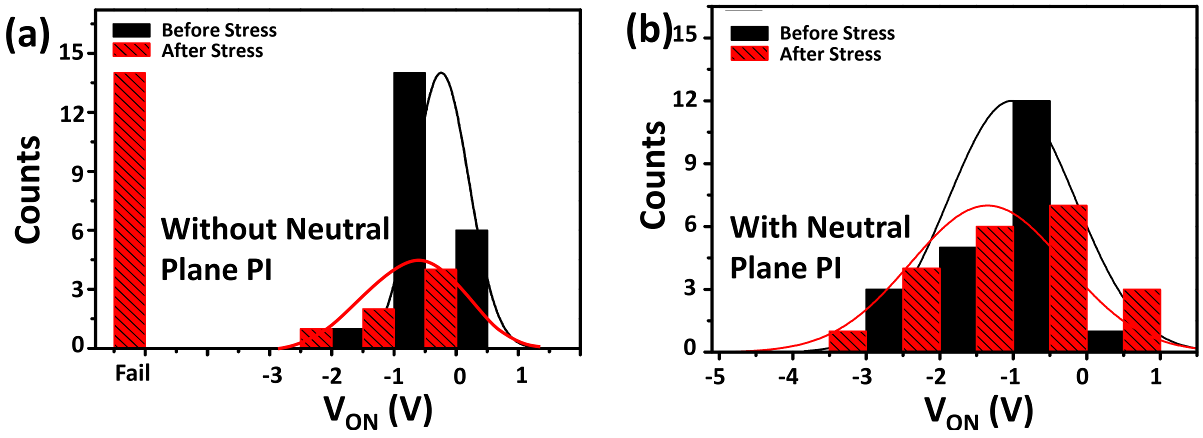

Figure S2. Histograms of the turn on voltage (V_{ON}) of TFTs (a) without (b) with the neutral plane PI film, before and after applying extreme bending stress. Bending radius = 0.25 mm and all TFTs have channel width (W) = 20 µm and channel length (L) = 6 µm. In (a), TFTs that have experienced breakdown due to cracks are labeled "Fail" and these are the TFTs that are no longer operational. The ones that are still functional, but have undergone negative ΔV_{ON}, have V_{ON} ranging from -2.5 to 0 V. In (b), all TFTs are operational.

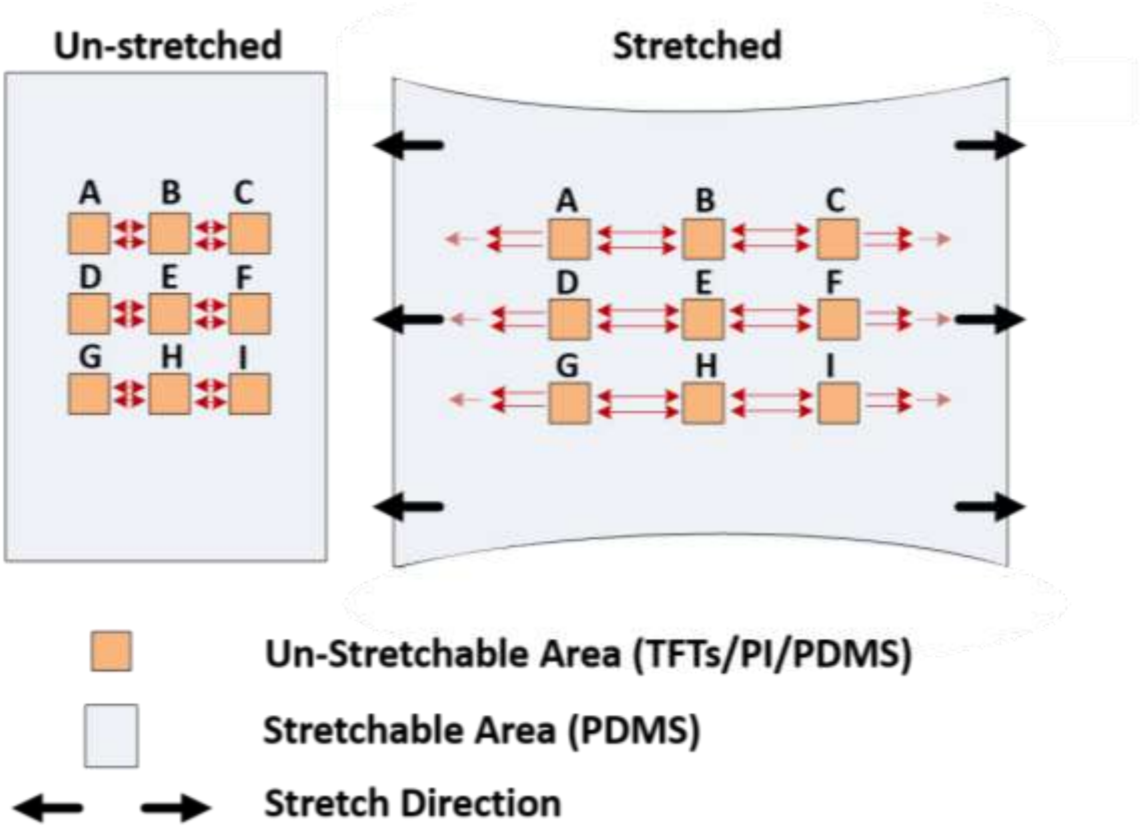

Figure S3. Stretch mechanics. The area of the sample that contains PDMS only is the area that stretches, while the area containing the TFT islands in un-stretchable.

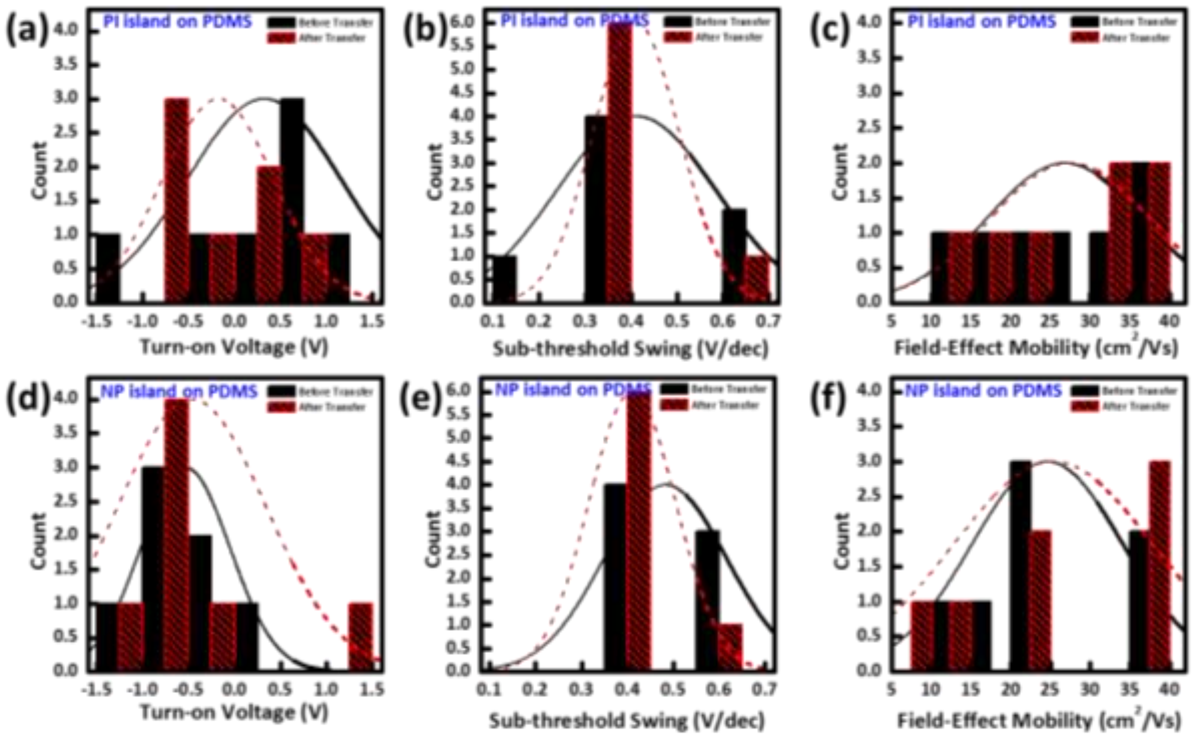

Figure S4. Comparison of turn-on voltage (V_{ON}), sub-threshold voltage swing (SS) and field-effect mobility (μ_{FE}) of TFTs before and after being transferred to PDMS as islands (a-c) without the neutral plane PI and (d-f) with the neutral plane PI. The black solid lines and black solid bars represent the characteristics before transfer to the PDMS. The red dashed lines and red striped bars represent the characteristics after transfer to the PDMS. All performance parameters of TFTs in the neutral plane show significantly smaller compared to those of the TFT that are not in the neutral plane after transfer to PDMS.
